# Hidden biodiversity in an ancient lake: phylogenetic congruence between Lake Tanganyika tropheine cichlids and their monogenean flatworm parasites

**DOI:** 10.1038/srep13669

**Published:** 2015-09-03

**Authors:** Maarten P. M. Vanhove, Antoine Pariselle, Maarten Van Steenberge, Joost A. M. Raeymaekers, Pascal I. Hablützel, Céline Gillardin, Bart Hellemans, Floris C. Breman, Stephan Koblmüller, Christian Sturmbauer, Jos Snoeks, Filip A. M. Volckaert, Tine Huyse

**Affiliations:** 1 Laboratory of Biodiversity and Evolutionary Genomics, Department of Biology, University of Leuven, Ch. Deberiotstraat 32, B-3000 Leuven, Belgium; 2 Biology Department, Royal Museum for Central Africa, Leuvensesteenweg 13, B-3080 Tervuren, Belgium; 3 Department of Botany and Zoology, Faculty of Science, Masaryk University, Kotlářská 2, CZ-611 37 Brno, Czech Republic; 4 Institute of Marine Biological Resources and Inland Waters, Hellenic Centre for Marine Research, 46.7 km Athens-Sounio Avenue, PO Box 712, Anavyssos GR-190 13, Greece; 5 Institut des Sciences de l'Évolution, IRD-CNRS-Université Montpellier 2, CC 063, Place Eugène Bataillon, F-34095 Montpellier cedex 05, France; 6 Institute of Zoology, University of Graz, Universitätsplatz 2, A-8010 Graz, Austria

## Abstract

The stunning diversity of cichlid fishes has greatly enhanced our understanding of speciation and radiation. Little is known about the evolution of cichlid parasites. Parasites are abundant components of biodiversity, whose diversity typically exceeds that of their hosts. In the first comprehensive phylogenetic parasitological analysis of a vertebrate radiation, we study monogenean parasites infecting tropheine cichlids from Lake Tanganyika. Monogeneans are flatworms usually infecting the body surface and gills of fishes. In contrast to many other parasites, they depend only on a single host species to complete their lifecycle. Our spatially comprehensive combined nuclear-mitochondrial DNA dataset of the parasites covering almost all tropheine host species (N = 18), reveals species-rich parasite assemblages and shows consistent host-specificity. Statistical comparisons of host and parasite phylogenies based on distance and topology-based tests demonstrate significant congruence and suggest that host-switching is rare. Molecular rate evaluation indicates that species of *Cichlidogyrus* probably diverged synchronically with the initial radiation of the tropheines. They further diversified through within-host speciation into an overlooked species radiation. The unique life history and specialisation of certain parasite groups has profound evolutionary consequences. Hence, evolutionary parasitology adds a new dimension to the study of biodiversity hotspots like Lake Tanganyika.

Elucidating speciation mechanisms is considered crucial for understanding the dynamics and function of biodiversity. Alternative speciation modes, such as allopatric, sympatric and parapatric speciation are increasingly understood with the help of phylogenetics^1^. A special process in this field is adaptive radiation, the phenomenon in which rapid speciation is combined with niche differentiation of the evolving species. Studying radiations has proven to be particularly promising to shed light on the causes and mechanisms driving speciation especially when dealing with species confined to a relatively closed system such as lakes^2^. One of the most prolific vertebrate radiations are the cichlid fishes (Teleostei, Cichlidae) of the East African Great Lakes^3^.

Lake Tanganyika, the oldest and deepest of these lakes, harbours the genetically and phenotypically most diverse cichlid community of these African lakes^4^. Its cichlid assemblage is subdivided into 12 to 17 mostly endemic tribes^5^. One of these tribes, the monophyletic Tropheini, is phylogenetically nested within the tribe Haplochromini and represents the sister group of the species flocks of Lake Malawi and the Lake Victoria region, and of several East African riverine lineages^6^. Tropheini consists of 23 endemic nominal species. Although considerable knowledge gaps exist regarding their taxonomy and distribution^7^, their phylogeny is well-resolved and updated^8^,^9^. Most species are adapted to rocky shores, and representatives of most genera occur sympatrically^7^,^8^. Tropheini contains generalist as well as specialist species that exhibit variable levels of genetic and phenotypic structuring, related to differences in habitat preference, dispersal ability and territoriality^8^. All these factors sparked substantial scientific interest and rendered the Tropheini radiation a “natural experiment” for species formation.

However, regardless of this showcase of biodiversity, the most spectacular radiations are found among parasites^10^. Mutual evolutionary pressures maintain genetic diversity in host and parasite, and fuel the rate of genetic diversification^11^. Moreover, the availability of numerous niches across a host’s body is an additional factor fostering parasite within-host diversification^12^ and hence speciation. Organisms with a parasitic lifestyle account for most of Earth’s biodiversity^13^. However, biodiversity studies tend to focus on conspicuous faunas, ignoring the vast biomass and species-richness of helminths and other less sizeable animals^14^,^15^. As such, the potential to understand speciation through the study of parasite evolution remains almost unexplored^12^,^16^ and the contribution of parasites to the species richness of the African Great Lakes has remained largely overlooked^17^,^18^.

We combine speciation research on cichlid hosts and their monogenean flatworm parasites. Monogeneans are mostly ectoparasites of cold-blooded aquatic or amphibious vertebrates although some infect aquatic invertebrates or exhibit an endoparasitic lifestyle^19^. Cichlid monogeneans provide a good model for elucidating parasite speciation^20^,^21^. Their direct (single-host) life cycle makes them particularly interesting, as it may be difficult to discern host factors that influence parasite evolution for parasites with an intermediate host^22^. Previous studies on Lake Tanganyika monogeneans uncovered a diverse and largely endemic fauna belonging to *Gyrodactylus* von Nordmann, 1832 and *Cichlidogyrus* Paperna, 1960^18^,^23^,^24^,^25^. The latter gill parasites represent the most abundant and prevalent monogenean genus on Tanganyika cichlids^23^. In most tropheine cichlid populations screened to this end, over two-thirds of fish individuals were infected by representatives of this genus^26^,^27^,^28^. Eggs of *Cichlidogyrus* develop and hatch on the bottom, after which a free-living ciliated larvae infects a host fish^29^.

We want to understand speciation by reconstructing the phylogenetic history of parasites belonging to *Cichlidogyrus*, retrieved from almost all tropheine host species with nuclear ITS rDNA and mitochondrial COI sequences. Taxonomic coverage is important in phylogenetic studies in general^1^ and in cophylogenetic work in particular^30^,^31^. The taxonomical and phylogenetic background that exists for the tropheine hosts (see above) is essential for a successful analysis of parasite diversification^31^. (i) We hypothesize that species of *Cichlidogyrus* infecting tropheine cichlids are host-specific, with a higher species richness on more stenotopic hosts. The generality of these patterns will be assessed to complement the few morphology-based reports^23^,^28^. (ii) The morphology of *Cichlidogyrus* suggests an influence of tropheine phylogeny on host choice^24^ and therefore we hypothesize that host and parasite phylogenies are to a certain extent congruent. However, following the results of Mendlová *et al.*^32^ for West African species of *Cichlidogyrus*, we expect that other speciation modes have also contributed to parasite diversification. Possible mechanisms of species formation in parasites include host-switching (ecological transfer between host species), cospeciation (concomitant speciation of host and parasite), duplication (within-host parasite speciation) and sorting (parasite extinction).

## Results

### Sequence diversity and host-specificity

**Figure 1:**
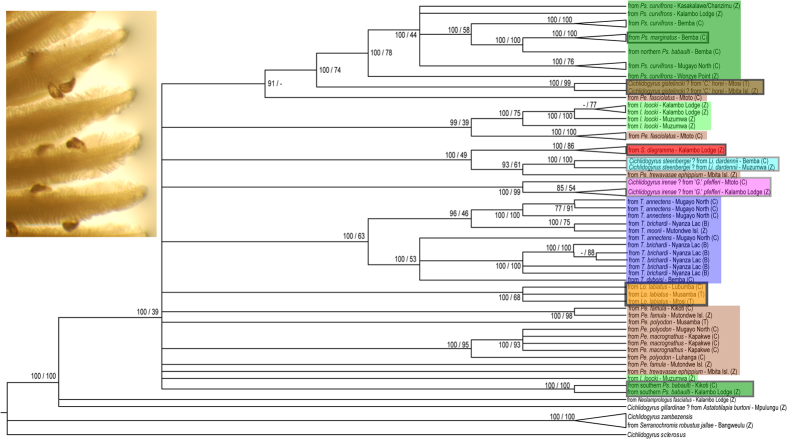
Consensus cladogram of flatworms belonging to the genus *Cichlidogyrus* infecting Lake Tanganyika cichlids. Cladogram based on the combined nuclear ITS-1, 5.8S rDNA, ITS-2 and mitochondrial COI sequences of *Cichlidogyrus* parasitizing Lake Tanganyika tropheine cichlids and the outgroups mentioned in Table 2. Statistical support is shown as posterior probability under BI/ML bootstrap. Clades that neither yield a support value of 85 nor of 70 under BI or ML, respectively, were collapsed; “–” indicates that a clade was not recovered in a particular analysis. Tip labels indicate host species with sampling locality and country (C: Democratic Republic of Congo; B: Burundi; T: Tanzania and Z: Zambia) and are coloured according to host genus consistent with Fig. 4. Monophyletic assemblages infecting one host species are boxed. Inlet: *Cichlidogyrus* parasites (300–400 μm in length) on the gills of *Sarotherodon melanotheron* Rüppell, 1852 (photograph taken by author A.P.).

The dataset based on a rDNA fragment of 1399 bp length contained 82 haplotypes and 575 variable sites, of which 323 were parsimony-informative. As two rDNA sequences each from parasite of *Petrochromis trewavasae trewavasae* and from *Petrochromis trewavasae ephippium* and three from parasites of *Tropheus brichardi* differed to the extent that meaningful alignment was hampered, they were excluded from further analyses. For COI, the dataset amounted to 583 bp, 73 haplotypes, 278 variable sites and 230 parsimony-informative sites. The concatenated dataset included 62 haplotypes and totaled 1935 nucleotide positions. This alignment contained 621 bp of ITS-1, 157 bp of 5.8S rDNA, 574 bp of ITS-2 and 583 bp of COI. There were 861 variable sites, of which 659 parsimony-informative. Table 1 provides an overview of the number of sequences and unique haplotypes retrieved for parasites of each host species, of corrected pairwise genetic distances between parasites of the respective host species, and of the estimated number of species sampled based on the species-level cut-offs proposed for the respective nuclear and mitochondrial sequences (see Materials and Methods). Our largest ITS rDNA dataset covers parasites of *Lobochilotes labiatus*, *Simochromis diagramma* and ‘*Ctenochromis*’ *horei*. Using ITS based species richness estimates, these cichlids harbor seven, three and one species of *Cichlidogyrus*, respectively. Identical parasite haplotypes were consistently retrieved from conspecific hosts. This host-specificity pattern is stronger than obvious from the phylogenetic tree (Fig. 1) as most haplotypes represent multiple individuals (Table 2).

### Phylogenetic analyses

Tree reconstruction on the basis of the concatenated dataset (Fig. 1) showed that all Tanganyika monogeneans are grouped in a well-supported clade. In the tree, the parasites of the following taxa are grouped together: the non-tropheine Tanganyika endemic *Neolamprologus fasciatus*, the haplochromine *Astatotilapia burtoni*, which is endemic to the Tanganyika Basin but not to the lake proper, and the Tropheini. Within this group, *Cichlidogyrus* infecting tropheines is supported as a monophyletic group. *Cichlidogyrus sclerosus* and its congener *C. zambezensis*, the latter hosted on the riverine haplochromine *Serranochromis robustus jallae*, are clearly separated from the Tanganyikan parasites. Well-supported clusters of *Cichlidogyrus* are organised according to host species. Irrespective of sampling locality, ‘*Ctenochromis*’ *horei*, ‘*Gnathochromis*’ *pfefferi*, *Limnotilapia dardennii*, *Lobochilotes labiatus*, *Simochromis diagramma* and southern *Pseudosimochromis babaulti* all harbour monophyletic parasite clades. The parasites of *Ps. curvifrons* are paraphyletic with respect to the lineages infecting northern *Ps. babaulti* and *Ps. marginatus*. This is in agreement with the hosts’ affinities within one genus and with the recent removal of *Ps. babaulti* and *Ps. marginatus* from *Simochromis*^25^. The *Tropheus* parasites cluster at the host genus level. The clades of *T. annectens* and *T. brichardi* parasites do not strictly follow host species boundaries. However, haplotypes were never shared between *Tropheus* species. *Petrochromis* does not host monophyletic parasite assemblages, which is in agreement with its paraphyly and need for taxonomic revision^8^,^24^.

**Figure 2:**
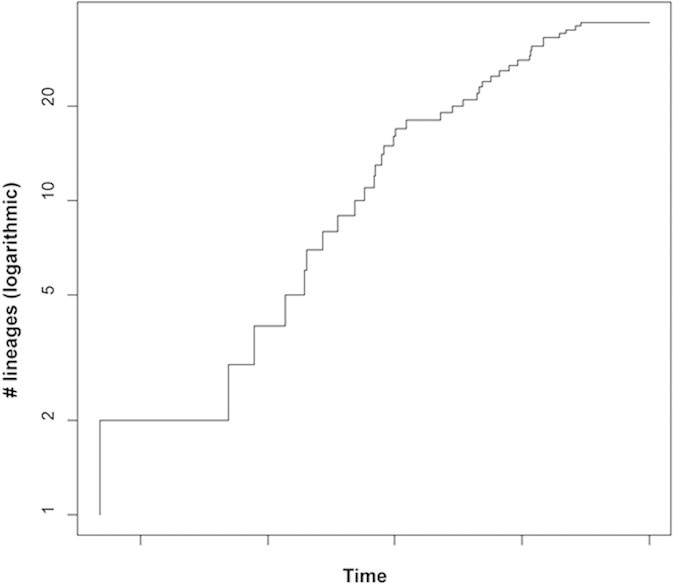
Lineages-through-time plot for *Cichlidogyrus* parasites of Tropheini. Lineages-through-time plot based on an ultrametric Bayesian ITS rDNA tree, constructed under a relaxed clock model, of *Cichlidogyrus* living on tropheine hosts; x-axis: time; y-axis: number of lineages (logarithmic scale).

A decrease in speciation rate towards the present can be observed on the LTT plot (Fig. 2). A positive value (17.41) for the difference in AIC score between the best-fit rate-constant and rate-variable model indicates that the speciation rate of *Cichlidogyrus* changed with time. This difference was significant as it outnumbered all differences in AIC scores comparing the same models for 5000 randomly generated trees of the same size.

### Cophylogenetic analyses

**Figure 3:**
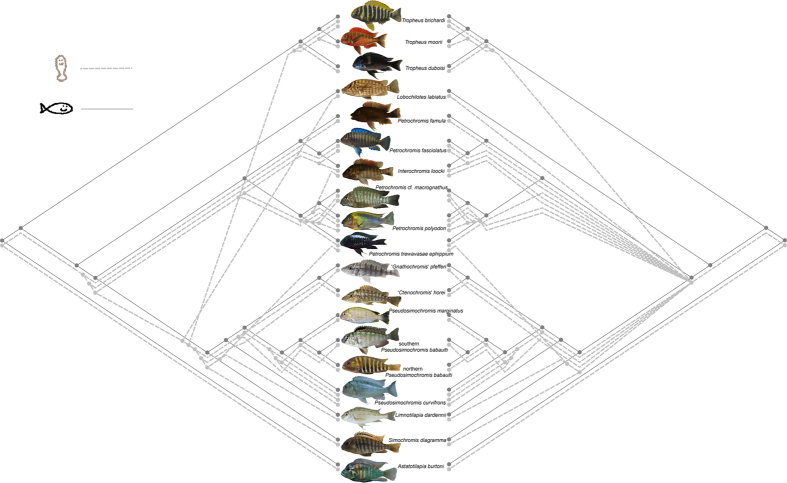
Co-phylogenetic reconciliations of *Cichlidogyrus* and Tropheini trees. CoRe-Pa reconciliations of Tropheini (based on AFLP as published by Koblmüller *et al.*^8^) and *Cichlidogyrus* (based on the concatenated nuclear-mitochondrial dataset) trees. Left: second best reconciliation based on a fully resolved parasite ML tree; right: best reconciliation based on a parasite ML tree where nodes with bootstrap support under 70 were collapsed. Branches and tips represent hosts (dark gray) or parasites (dashed/light gray). Drawings made by author T.H.; photographs taken by authors P.I.H. (*I. loocki*, *L. labiatus*, *Pe. famula*, *T. moorii*), M.P.M.V. (*Ps. curvifrons*) and M.V.S. (*L. dardennii*) and reproduced with kind permission from Radim Blažek (‘*C.*’ *horei*, ‘*G.*’ *pfefferi*, northern *Ps. babaulti*, *S. diagramma*, *T. duboisi*) and Ad Konings (*A. burtoni*, *Pe. fasciolatus*, *Pe. macrognathus*, *Pe. polyodon*, *Pe. trewavasae ephippium*, *Ps. marginatus*, southern *Ps. babaulti*, *T. brichardi*).

The two best solutions proposed by the CoRe-Pa software, either based on a resolved parasite tree or a parasite tree with a basal polytomy, are presented in Table 3. The best solution for the resolved tree and the second best solution in the polytomy case invoked no host-switching and an unrealistically high cost for it, in comparison to the costs inferred for other events. These scenarios are not biologically plausible. Cost schemes should not only be judged on a purely statistical basis but also include the biological context^33^. Hence these reconstructions were not further considered and, in case of the resolved tree scenario, replaced by the second best proposal (Fig. 3). The two retained solutions, with and without a polytomy in the parasite tree, proposed comparable numbers for all events, with a high number (33–40) of sortings, similar frequencies of cospeciation (11–13) and within-host speciation events (12-11) and a low number of host-switches (4-3) (Table 3; Fig. 3). In the software package TreeMap the inferred number of 12 cospeciation events was found statistically significant at a level of 0.05, because only 421 out of 10^4^ random reconciliations included 12 or more cospeciation events. This indicates topological congruence between host and parasite trees. Overall congruence between the host and parasite mitochondrial genotypes was significant in a distance-based cophylogenetic analysis (*P* < 0.01) although only 11 out of 47 links were reported as significant at a level of 0.05.

## Discussion

The well-studied cichlid tribe Tropheini of Lake Tanganyika, a lineage of endemic and mainly rock-dwelling species, was used as a framework to study parasite diversity and speciation. Phylogenetic reconstruction of its monogenean parasites belonging to *Cichlidogyrus* covered nearly all nominal tropheine host species and resulted in a clear pattern of host-specificity and congruence between host and parasite trees. We explore how parasite diversity relates to the biology of the respective host species, and how parasite speciation mechanisms relate to the radiation within Tropheini.

Representatives of *Cichlidogyrus* are abundant on tropheine hosts: our sampling shows a picture throughout the tribe’s populations, similar to previous case studies^26^,^27^,^28^, of two-thirds to all of the hosts infected (unpublished data). Many species within the genus infect just one (or a set of closely related) host species^21^. However, host-specificity varies among species and lineages of *Cichlidogyrus*, and the degree of host-specificity may correlate with the biology of the host^34^. In Lake Tanganyika, a rather generalist species of *Cichlidogyrus* infects pelagic bathybatines^18^. Conversely, based on the few morphology-based case-studies, representatives of *Cichlidogyrus* seemed to be host-specific on littoral Tanganyika cichlids (overview in Pariselle *et al.*^18^). We confirm this host-specificity genetically for parasites belonging to *Cichlidogyrus* of the entire tribe Tropheini. Conspecific cichlid populations host the same parasite species even when geographically separated by hundreds of kilometers. This is clearly exemplified by the monophyletic and monospecific parasite clades of ‘*C.*’ *horei*, ‘*G.*’ *pfefferi*, *L. dardennii* and southern *Ps. babaulti* (Table 1; Fig. 1). Conversely, sympatric host species had their unique set of parasite species. On several localities in the D.R. Congo and Zambia, sampling comprised representatives of the four main clades within Tropheini, namely *Lobochilotes*, *Petrochromis*/*Interochromis*, *Tropheus* and the ‘substrate dwellers’ including ‘*Ctenochromis*’, ‘*Gnathochromis*’, *Limnotilapia*, *Pseudosimochromis* and *Simochromis*^8^. Their parasite fauna never overlapped. As eggs of *Cichlidogyrus* develop away from their parental host and infective larvae have to actively colonize a new fish, each parasite individual in the survey may be considered an independent observation. Hence there is little chance of overestimating host-specificity, unlike species with clonal reproduction on the host (e.g. within *Gyrodactylus*^35^).

This consistent host-specificity is remarkable in view of tropheine ecology; with many species sympatrically inhabiting shallow rocky habitat, opportunities for parasite transfer are plenty. Monogeneans recognize their host using the species-specific chemical and physical properties of the fish’ integument^36^. Interindividual and interspecific variation in chemical cues characterize cichlids, as evidenced by tests for olfaction-based mate recognition^37^. Chemical cues emitted by the tropheine hosts might hence explain how flatworms belonging to *Cichlidogyrus* discern between host species. The life history of *Cichlidogyrus* seems to select for successful colonization through host specialisation, as larvae are shortlived and therefore have to find a suitable host soon. Moreover they do not have a second chance because they cannot switch hosts after attachment^29^. It should be noted that factors other than colonization of the host may also explain host-specificity. For example, differential survival after reaching an ant host colony is considered important in the specificity of “cuckoo species” of myrmecophilous lycaenid butterflies^38^. Competition can also mediate host-specificity^39^. Anyhow, the narrow host-range of *Cichlidogyrus* from the tropheine system is striking, given that some congeners display a much wider host range, both within^18^ and outside Lake Tanganyika^34^,^40^. More specific monogeneans tend to be found on larger-bodied or longer-lived fishes and their specialisation is considered to be a consequence of higher predictability of host resources^41^,^42^. While Mendlová and Šimková^34^ did not find evidence of this predictability hypothesis in *Cichlidogyrus* with regard to host body size or longevity, we assume that Lake Tanganyika tropheine littoral cichlids are predictable resources as regards their ecology because their abundance and species-richness are higher than for cichlids in the pelagic realm^5^,^17^. It has been observed in many systems that abundant hosts harbor more specialised parasite species^43^.

Our estimates of species richness (Table 1) have to be regarded with caution because sample size plays a role when estimating species numbers. The risk of underestimating parasite diversity is especially valid in rarely sampled host species. However, for the tropheine hosts of which the species of *Cichlidogyrus* are well characterized morphologically, ITS based estimates correspond to the number of formally described parasite species: one in ‘*Ctenochromis*’ *horei*, ‘*Gnathochromis*’ *pfefferi* and *Limnotilapia dardennii*^44^ and three in *Interochromis loocki*^24^ and *Simochromis diagramma*^25^. By focusing on the best-sampled species, a pattern emerges. A stenotopic host such as *Lobochilotes labiatus* harbors more species of *Cichlidogyrus* than eurytopic cichlids such as *Simochromis* species and ‘*C.’ horei*^8^. This matches with the morphology-based observation of Grégoir *et al.*^28^, which was based on just two tropheine species. Hence, as suggested by Pariselle *et al.*^18^, host isolation or migration influences the species richness of the *Cichlidogyrus* community. Many factors were mentioned to determine the number of congeneric parasites a host supports, but the evolutionary mechanisms remain poorly understood^45^. Isolation among host populations is suggested as a strong driver of parasite genetic structuring^16^,^46^ and is here proposed to also promote parasite speciation.

Parasites provide useful complementary data on their fish host, e.g. in elucidating their hosts’ biogeography, identification or phylogeny^47^. The affinities between representatives of *Cichlidogyrus* in closely related hosts corroborate recent findings on the phylogenetic relationships among the Tropheini in several aspects. Firstly, the monophyletic clustering of *Tropheus* parasites includes parasites found on *T. duboisi*. The position of this host species within *Tropheus* was confirmed only recently^8^. Secondly, *Ps. marginatus* parasites are phylogenetically nested within those of the closely related *Ps. curvifrons*, corroborating their shared parasite species^25^. Thirdly, although they also share a species of *Cichlidogyrus*^25^, northern and southern *Ps. babaulti* are also infected by monogeneans which are not closely related. Hence parasite data agree with the morphological and genetic differentiation of geographically separated *Ps. babaulti* populations. This corresponds with the historical division of the Lake in subbasins, the influence of which on cichlid diversity is well-documented^48^.

The significant congruence between host and parasite phylogenies in distance-based and topology-based cophylogenetic analysis suggests that cospeciation played an important role in the diversification of the parasite fauna of the Tropheini. Although host-specificity may promote cospeciation^49^, it does not preclude speciation through host-switching^50^. Since the seminal paper of Hafner *et al.*^51^ which showed cospeciation between gophers and their ectoparasitic lice, cospeciation has rarely been demonstrated. Many empirical studies have shown that the combination of host and parasite traits and biogeography led to little or no congruence between host and parasite phylogenies, even when there is host-specificity (e.g. for *Rhabdomys* four-striped mice and *Polyplax* sucking lice^52^). Even in the case of phylogenetic congruence, other factors have been shown to be stronger drivers of speciation than coevolutionary interactions, such as geographic isolation (yuccas and associated prodoxid moths^53^) or phylogenetically constrained host-switching (gobies and *Gyrodactylus*^54^). For several systems, including *Cichlidogyrus* of West African cichlids, host-switching and duplication were suggested to be the underlying mechanisms of parasite diversity^32^. The difference with our results could be explained by two factors. West African cichlids are infected by a combination of specialist and more generalist monogeneans, and the ecological differences between the lacustrine tropheines and the more generalist cichlids included in Mendlová *et al.*^32^ are considerable. Topological reconciliations propose that cospeciation and duplication events are three to four times as frequent as host-switches in the *Cichlidogyrus*-Tropheini system, and that the number of parasite extinctions is high (Table 3). Frequent extinction is likely in the case of overdispersion, which is common in Monogenea^20^. In addition, the often small and fluctuating population sizes of monogeneans^55^ may promote extinction. The fact that the outcome is very similar for fully resolved and unresolved parasite trees (Table 3; Fig. 3) suggests that the proportions are robust and not an artefact of poor phylogenetic resolution. In order to discriminate cospeciation from preferential host-switching, an absolute timeframe is required to establish temporal congruence^54^,^56^. Most parasite genetic distances between host species range between 2 and 7% (ITS). Using the 2.4 Mya estimate of Koblmüller *et al.*^8^ for the most recent common ancestor of Tropheini, this would translate into an ITS mutation rate of *Cichlidogyrus* of 0.4–1.5% my^−1^. This is lower than the rate of 5.5% my^−1^ calculated for *Gyrodactylus*^57^ but this monogenean has a much shorter generation time^35^, which likely results in a higher mutation rate^58^. Therefore, four conclusions come to mind. (1) Divergence within Tropheini and the associated *Cichlidogyrus* fauna was probably concomitant. (2) The basal polytomy in the parasite tree (Fig. 1) represents a true (“hard”) polytomy, congruent with the rapid radiation of the host which led to a similar polytomy^8^. (3) It is likely that the diversification of these flatworms happened within the confines of Lake Tanganyika in view of the monophyly of the ingroup. (4) Simultaneous diversification of tropheine cichlids and their parasites belonging to *Cichlidogyrus* might explain the decrease in parasite speciation rate towards the present (Fig. 2), which is indicative of a radiation event^1^.

Cospeciation has rarely been observed in fish parasites in general^22^,^33^,^59^ or in monogeneans in particular^60^. In terrestrial systems it is in many instances a by-product of restricted contact between host species (phthirapteran chewing lice of geomyid pocket gophers^51^ or of seabirds^61^) or predominantly vertical transmission (*Buchnera* symbiotic bacteria of *Uroleucon* aphids^30^). Neither of them apply to representatives of *Cichlidogyrus* infecting the many sympatrically occurring^8^ tropheine cichlids. Our survey covers aquatic hosts occurring in the same lentic microhabitats, with parasites that have a free-living and actively recolonizing larval stage, hence offering plenty of opportunities for host-switching. However, the colonisation mode of the parasite, with a single attachment event to the host, a short survival time away from the host, and sufficient access to the typical host species in the littoral habitat, seems to select for a specific host choice and against ecological transfer. Both the topology-based phylogenetic analysis and the monophyletic host-associated clusters point to the equally important role of within-host speciation. Its importance has been reported in other dactylogyridean monogeneans, e.g. on West African cichlids^32^, European cyprinids^62^ and Asian pangasiids^63^. Hence, we propose that the monogenean fauna diversified as a result of reproductive isolation following host speciation, in combination with within-host duplication resulting in higher parasite than host diversity. When parasites on a shared host species are sister taxa like in the present case, Poulin^64^ suggests that they may be named parasite species flocks. Hence, we propose that the *Cichlidogyrus* fauna on tropheine cichlids is an overlooked case of the numerous invertebrate radiations of Lake Tanganyika^17^.

## Methods

### Sampling and data collection

**Figure 4:**
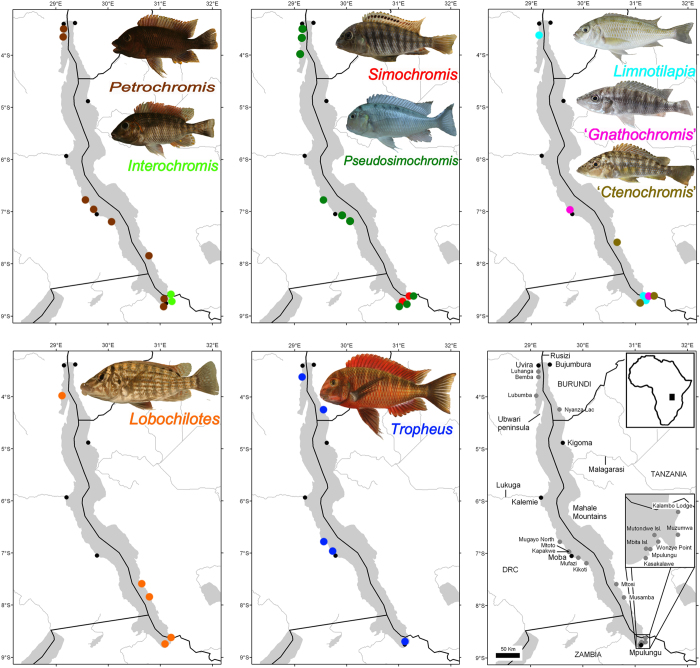
Lake Tanganyika localities sampled for monogenean cichlid parasites belonging to *Cichlidogyrus*. Colour codes refer to the respective host genera; the bottom right map details the sampling localities and major cities. For details, see Table 2. Photographs were taken by authors P.I.H. (*I. loocki*, *L. labiatus*, *Pe. famula*, *T. moorii*), M.P.M.V. (*Ps. curvifrons*) and M.V.S. (*L. dardennii*) and reproduced with kind permission from Radim Blažek (‘*C.*’ *horei*, ‘*G.*’ *pfefferi*, *S. diagramma*). Map created using ArcMap v.10 and reproduced with kind permission by Tobias Musschoot.

*Cichlidogyrus* specimens were collected on 18 of the 23 nominal species of Tropheini. Nominal species excluded are *Tropheus kasabae* Nelissen, 1977, a junior synonym of *T. moorii*^65^; *T. polli* Axelrod, 1977, a junior synonym of *T. annectens*^65^; *Petrochromis horii* Takahashi & Koblmüller, 2014, which was unknown at the time of sampling; *Petrochromis orthognathus* Matthes, 1959 and *Simochromis margaretae* Axelrod and Harrison, 1978. The latter species is known only from four museum specimens, none of which suited for molecular analyses; inspection of two of these individuals did not yield gill monogeneans. We distinguish between northern and southern *Ps. babaulti*. Indeed, the southern populations form a separate clade^8^ and were classified at the time of sampling as a separate species: *Ps. pleurospilus* (Nelissen, 1978). The latter species was only recently synonymized with *Ps. babaulti*^25^. Hence, host taxon sampling is almost as exhaustive as possible. Given the position of the Tropheini within the haplochromines (see above), two haplochromines were included to use their parasites belonging to *Cichlidogyrus* as outgroup. These are *Astatotilapia burtoni*, a derived haplochromine which occurs in Lake Tanganyika tributaries, and a more basal representative of the Haplochromini, *Serranochromis robustus jallae* from southern Africa. In addition, the lamprologine *Neolamprologus fasciatus* was sampled as it shares the rocky littoral habitat with many tropheines. Figure 4 and Table 2 provide a detailed overview of species and locations from which samples were retrieved.

Cichlids were collected in the rocky littoral of Lake Tanganyika using gill nets. Sampling protocols were approved by the following competent national authorities, and carried out in accordance with research permit no. 2007-258-CC-2006-151 from the Tanzania Commission for Science and Technology (COSTECH); the memorandum of understanding between the Karl-Franzens University of Graz, the University of Zambia and the Department of Fisheries, Zambian Ministry of Agriculture and Co-operatives; and mission statement no. 013/MNRST/CRHU/2010 from the Ministère de la Recherche Scientifique et Technologique–CRH-Uvira. Newly collected fish were kept alive in aerated tanks until they were sacrificed by severing the spinal cord or with an overdose of MS-222. They were identified to species level *in situ* and in the laboratories of the RMCA, where host vouchers are kept (Table 4). Host fish or their branchial arches were fixed and stored in pure ethanol. Gills were inspected for monogeneans under an Olympus SZX12 stereomicroscope. Parasites were isolated with a dissection needle and stored in 5 μl of milli-Q H_2_0 at −20 °C awaiting further processing. A small number of flatworms were stored in the field on FTA Classic Cards (Whatman). In total, sequences were obtained from 220 parasite specimens, retrieved from 84 cichlid individuals. DNA extraction, PCR amplification and sequencing followed Vanhove^23^ (Supplementary Methods S1). Table 4 shows the GenBank accession numbers of the parasite sequences obtained.

### Phylogenetic analyses of parasites belonging to *Cichlidogyrus*

The closest BLAST hit from GenBank, the complete ITS-1 sequence of *Cichlidogyrus sclerosus* Paperna and Thurston, 1969 (DQ537359) was included as additional outgroup for rooting. Sequence alignment was performed by MUSCLE v.3.8^66^ under default distance measures and sequence weighting schemes. The resulting alignments were visually inspected and improved in MEGA v.5^67^. In the case of COI, alignment was straightforward as there were no gaps and translation into amino acids (using the echinoderm and flatworm mitochondrial code) did not result in nonsense or stop codons. These nuclear and mitochrondrial datasets were used separately for an assessment of genetic diversity. jModelTest v.0.1.1^68^ was used to select the optimal molecular evolution model starting from a maximum likelihood (ML) optimized tree. Based on the corrected Akaike information criterion (AICc), the TVM + Γ model was selected for the nuclear alignment and the TIM2 + I + Γ model for the mitochondrial dataset (with gamma shape parameter of 0.40 for ITS rDNA and 0.11 for COI). Gamma-corrected pairwise genetic distances were calculated in PAUP* v.4.01b (Swofford, 2001, Sinauer Associates). As a rough estimate of the species diversity contained in the sample, the number of haplotypes displaying at least 1% (for ITS rDNA) and 2% divergence (for COI) was determined. This follows the ITS divergence cut-off proposed to match morphospecies boundaries in the best-studied monogenean, *Gyrodactylus*^57^, and the threshold of sequence divergence between species commonly used in barcoding^69^.

For tree reconstruction, a concatenated dataset was built on the basis of the specimens that yielded both nuclear and mitochondrial sequences. For an assessment of the phylogenetic content of the dataset, we performed a likelihood mapping analysis based on quartet puzzling^70^ implemented in TREE-PUZZLE v.5.2^71^. In this combined alignment, the proportion of fully resolved quartets was 85.2%, with 9.4% partly resolved and 5.5% unresolved. In view of its relatively high phylogenetic content and the use of independently evolving (unlinked) markers, such concatenated nuclear-mitochondrial dataset allows for more robust (co-)phylogenetic hypotheses to be put forward (see also the recommendations by de Vienne *et al.*^56^).

Bayesian inference of phylogeny (BI) was carried out in MrBayes v.3^72^. Posterior probabilities were calculated over 10^7^ generations. Stationarity of the Markov chain was reached, as evidenced by a standard deviation of split frequencies of 0.008, by a potential scale reduction factor converging to 1 and by the absence of a trend in the plot of log-probabilities as a function of generations. The Markov chain was sampled with a frequency of 10^2^ generations; one-fourth of the samples were discarded as “burn-in”. A ML search was carried out in RAxML v.7.3.0^73^, assessing nodal support through 1000 bootstrap samples. The evolutionary model was optimized for each fragment separately, suggesting HKY + Γ for ITS-1, JC for 5.8S rDNA, TPM1uf + Γ for ITS-2 and GTR + Γ for COI. These models were substituted by GTR + Γ in RAxML and, in the case of ITS-2, also in MrBayes, as this was the implemented model with the best AICc score. In the latter software, all parameter estimates for the various sequence portions were unlinked.

To assess the rate of diversification as a function of time, we started from the largest dataset (ITS rDNA) in order to include a maximal sample size. Identical sequences were removed, as well as haplotypes differing less than 0.01, with the help of the CD-HIT Suite web server^74^, and additional manual removal in case of length differences. This sequence selection (see above for the rationale behind this cut-off) ensures a focus on speciation rather than on intraspecific variation. For this nuclear alignment, MEGA selected the HKY + Γ model as the optimal model of molecular evolution based on the Bayesian information criterion. Under this model, an ultrametric tree was built in BEAST v.1.8.1^75^ applying four rate categories with the initial and average value of the gamma shape parameter set to 0.29, under the Yule tree prior and an uncorrelated relaxed log-normal clock model. Indeed, a likelihood-ratio test conducted in TREE-PUZZLE had rejected the molecular clock hypothesis. The Markov Chain Monte Carlo run was run for 10^7^ generations with a sample frequency of 10^3^ generations; a burn-in of one-tenth was applied. Based on this tree, a lineages-through-time (LTT) plot was constructed in APE^76^. In view of the possibly ambiguous interpretation of the course of a LTT, LASER^77^ was used to quantify possible changes in diversification rate over time. This package compares the likelihood of data under models with a constant *versus* variable rate of diversification by contrasting the AIC score of the best-fit rate-constant model with that of the best-fit rate-variable model. To assess statistical significance, the difference in AIC scores between the best-fit rate-constant and rate-variables models was calculated for 5000 randomly generated trees, using the same set of models and the same tree size (37 terminal nodes).

### Cophylogenetic analyses

A range of methods exists to compare the phylogeny and divergence in host-parasite or other symbiotic systems, inferring the speciation patterns that contributed to the consistencies or inconsistencies in their evolutionary trajectories. Of these, methods reconciling host and parasite tree topologies are often considered to maximize cospeciation events, and to take topological congruence as an evidence for cospeciation, which is not always justified^56^. To minimize the risks of such assumptions, host and parasite tree were reconciled in the software package CoRe-Pa v.0.5^78^, checking 10^4^ cost sets using a simplex method on the quality function. This software carries out an event-based analysis. A major asset is its parameter-adaptive approach that allows for the automated estimation of proportional event costs, thus avoiding *a priori* cost assignment to the different categories of parasite speciation mechanisms^79^,^80^. Root-to-root mapping was enforced and the chronological consistency of events checked. To work with a fully resolved tree that best approaches the “species tree”, the ML parasite phylogram reconstructed by RAxML for the combined nuclear-mitochondrial dataset, was included in cophylogenetic analysis. The AFLP tree of Koblmüller *et al.*^8^ was coded with the help of TreeSnatcher^81^ to provide a host topology. The selection of optimal trees obviously entails some uncertainty for this topology-based analysis. Therefore, the analysis was repeated with a parasite phylogeny in which all nodes receiving less than 70% of bootstrap support were collapsed. As within-host speciation in terminal taxa can artificially infer cospeciations at the cost of additional duplications^56^,^63^, terminal monophyletic clades associated with a single host species were collapsed. For the same reason, the software was set not to bill an additional duplication for a host-switching event. Host and parasite tree were also reconciled in a heuristic search in TreeMap v.1.0a^82^. Because this topology-based software is known to maximize the number of cospeciation events, the number of the various speciation mechanisms that TreeMap proposes will not be taken into consideration. The test for the significance of the number of cospeciation events inferred is only considered as a measure of topological congruence rather than of cospeciation^56^. This was assessed by randomizing host and parasite topologies (10^4^ random trees used) under the proportion-to-distinguishable model.

Distance-based cophylogenetic analyses test for correlation between phylogenies without assuming congruence to be produced by cospeciation, and are hence considered less biased than topology-based methods^56^. Distance-based cophylogenetic analysis was carried out in Copycat v.1.14^83^ making use of AxParafit and AxPcoords^84^ using 9999 permutations under default settings. In this analysis, the independence between host and parasite patristic distances is tested. To this end, distance matrices were constructed with the help of T-rex^85^. For the hosts, the mitochondrial ND2 and control region data from Koblmüller *et al.*^8^ were used, as these mitochondrial fragments are better suited for distance calculations than AFLP data (long terminal and short internal branches in the AFLP tree). Because of the large number of insertions/deletions in the nuclear fragment which inevitably bias genetic distance estimates over the whole dataset, only all COI mitochondrial fragments, omitting the third codon position, were used to infer parasite patristic distances.

## Additional Information

**How to cite this article**: Vanhove, M. P. M. *et al.* Hidden biodiversity in an ancient lake: phylogenetic congruence between Lake Tanganyika tropheine cichlids and their monogenean flatworm parasites. *Sci. Rep.* **5**, 13669; doi: 10.1038/srep13669 (2015).

## Supplementary Material

Supplementary methods S1

## Figures and Tables

**Table 1:** Summary of the sequence dataset of *Cichlidogyrus* flatworms from Lake Tanganyika.

Host species	ITS rDNA			COI			estimated #species	
	#sequences	#haplotypes	distances	#sequences	#haplotypes	distances	Ziętara & Lumme	Hansen *et al*.
*C. horei*	30	4	0.1–0.7	2	2	11.6	1	2
*G. pfefferi*	5	3	0.1–0.3	3	3	0.5–2.2	1	2
*I. loocki*	8	3	1.3–2.0	5	5	0.3–17.8	3	3
*Li. dardennii*	5	3	0.1–0.3	3	3	0.5–17.0	1	2
*Lo. labiatus*	46	26	0.1–3.9	3	3	12.6–14.7	7	3
*Pe. famula*	6	3	0.2–1.5	3	3	6.5–16.8	2	3
*Pe. fasciolatus*	3	2	0.9	3	3	0.2–15.8	1	2
*Pe. macrognathus*	3	1	/	6	6	1.1–18.0	1	5
*Pe. polyodon*	8	6	0.6–4.3	5	5	1.8–19.3	5	4
*Pe. trewavasae trewavasae*	2*	/	/	5	5	0.4–20.1		
*Pe. trewavasae ephippium*	10 (+2*)	3	2.4–3.2	2	2	19.3	3	2
northern *Ps. babaulti*	2	1	/	3	2	0.7	1	1
southern *Ps. babaulti*	3	2	0.3	5	4	0.7–22.0	1	3
*Ps. curvifrons*	12	6	0.1–6.3	7	7	0.4–14.7	4	2
*Ps. marginatus*	13	5	0.1–2.5	2	2	0.2	2	1
*S. diagramma*	23	4	0.1–1.5	2	2	14.4	3	2
*T. annectens*	5	3	0.2–5.7	4	4	0.2–27.8	2	2
*T. brichardi*	6 (+3*)	2	3.4	9	6	0.7–18.4	2	2
*T. duboisi*	3	1	/	1	1	/	1	1
*T. moorii*	5	2	4.8	1	1	/	2	1
*A. burtoni*	/	/	/	2	1	/		1
*Se. robustus*	1	1	/	3	3	1.1–1.9	1	1
*N. fasciatus*	1	1	/	/	/	/	1	/

Gamma-corrected pairwise genetic distances (in %) between *Cichlidogyrus* haplotypes retrieved within respective host species, with indication of the number of sequences and unique haplotypes obtained for each sequenced region. The estimated number of *Cichlidogyrus* species per host species in this dataset is given according to the rules-of-thumb mentioned (for ITS) in Ziętara & Lumme^57^ and (for COI) in Hansen *et al.*^69^.

(*not included in the analysis–non-alignable).

**Table 2:** Overview of cichlids, parasites and locations sampled.

	LT																		BW
	DRC								B	T		Z							
	Luhanga, 27/3/2010 3°31′04′′S, 29°08′57′′E	Bemba, 26/3/2010 3°37′22′′S, 29°08′56′′E	Lubumba, 24/3/2010 3°58′54′′S, 29°06′32′′E	Mugayo North, 11/4/2010 6°46′42′′S, 29°33′30′′E	Mtoto, 15/4/2010 6°58′03′′S, 29°43′50′′E	Kapakwe, 15/4/2010 6°58′27′′S, 29°44′05′′E	Mufazi, 13/4/2010 7°05′12′′S, 29°54′45′′E	Kikoti, 20/4/2010 7°11′28′′S, 30°04′01′′E	Nyanza Lac, 2/5/2010 4°14'38" S, 29°33'17" E	Mtosi, 24/4/2008 7°35'27''S, 30°38´29′′E	Musamba, 25/4/2008 7°49'54′′S, 30°56´49′′E	Kalambo Lodge, 15–19/4/2008 8°37'22''S, 31°12'02''E	Muzumwa, 3/9/2011 8°42'06"S, 31°11'60''E	Wonzye Point, 12/4/2008 8°43'31''S, 31°08'00''E	Mutondwe Island, 11/4/2008 08°42'09S'', 31°07'12''E	Kalambo River Delta, Chipwa, 9/2011 8° 36' 6'' S, 31°11' 12'' E	Mbita Island, 9–10/4/2008 8°44'55''S, 31°05'28''E	Kasakalawe/Chanzimu, 13/4/2008 8°46'52''S,31°05'25''E	Fiwili, 18/7/2010 11°57'S 30°15'E
Tropheini																			
‘*C.*’ *horei* (Günther, 1894)										(1)3/1/1		(9)24/0/-					(1)3/1/1		
‘*G.*’ *pfefferi* (Boulenger, 1898)					(1)1/1/1							(3)4/2/2							
*I. loocki* (Poll, 1949)												(1)2/2/2	(2)6/3/3						
*Li. dardennii* (Boulenger, 1899)		(1)2/1/1										(1)2/0/-	(1)1/2/1						
*Lo. labiatus* (Boulenger, 1898)			(1)4/1/1							(1)2/1/1	(1)2/1/1	(5)36/0/-					(1)2/0/-		
*Pe. famula* Matthes and Trewavas, 1960								(1)1/1/1							(2)5/2/2				
*Pe. fasciolatus* Boulenger, 1914					(1)3/3/3														
*Pe. macrognathus* Yamaoka, 1983	(1)0/3/-					(1)3/3/3													
*Pe. polyodon* Boulenger, 1898	(1)1/1/1	(1)0/1/-		(1)1/2/1							(2)5/1/1						(1)1/0/-		
*Pe. trewavasae trewavasae* Poll, 1948								(3)2/5/2											
*Pe. trewavasae ephippium* Brichard, 1989															(1)6/0/-		(2)6/2/2		
northern *Ps. babaulti* (Pellegrin, 1927)		(1)2/3/2																	
southern *Ps. babaulti*							(2)0/2/-	(1)1/1/1				(2)2/2/2							
*Ps. curvifrons* (Poll, 1942)	(1)1/0/-	(1)3/2/2		(1)2/2/2								(2)4/1/1		(1)1/1/1				(1)1/1/1	
*Ps. marginatus* (Poll, 1956)		(1)2/2/2	(2)11/0/-																
*S. diagramma* (Günther, 1894)												(6)21/2/2					(1)2/0/-		
*T. annectens* Boulenger, 1900				(2)4/4/4		(1)1/0/-													
*T. brichardi* Nelissen and Thys van den Audenaerde, 1975									(4)9/9/6										
*T. duboisi* Marlier, 1959		(1)3/1/1																	
*T. moorii* Boulenger, 1898															(2)5/1/1				
Haplochromini																			
*A. burtoni* (Günther, 1894)																(1)0/2/-			
*Se. robustus* (Günther, 1864)																			(1)1/3/-
Lamprologini																			
*N. fasciatus* (Boulenger, 1898)												(1)1/0/-							

Numbers between brackets indicate the number of host fish specimens used; other numbers represent the number of *Cichlidogyrus* specimens sequenced as follows: number of nuclear ITS rDNA sequences obtained/number of mitochondrial COI sequences obtained/number of specimens of which both sequences were obtained. LT: Lake Tanganyika; BW: Bangweulu Wetlands; DRC: Democratic Republic of Congo; B: Burundi; T: Tanzania; Z: Zambia.

**Table 3:** Cophylogenetic reconciliations proposed by CoRe-Pa.

	Quality	Total cost	Cospeciation	Sorting	Duplication	Host-switch
Fully resolved	0.0054	0.61	12 (0.017)	51 (0.0040)	15 (0.013)	0 (0.97)
	**0.04**	**9.53**	**11 (0.25)**	**33 (0.074)**	**12 (0.20)**	**4 (0.47)**
Basal polytomy	**0.012**	**7.98**	**13 (0.16)**	**40 (0.051)**	**11 (0.19)**	**3 (0.60)**
	0.018	0.032	10 (0.000998)	65 (0.00017)	17 (0.00067)	0 (0.998)

Quality value, total value, and number of cospeciation, sorting, duplication and host-switch events invoked (with estimated cost in brackets) for the two best CoRe-Pa reconciliations of Tropheini (based on the AFLP markers as published by Koblmüller *et al.*^8^) and *Cichlidogyrus* (based on the concatenated nuclear-mitochondrial dataset) trees. Topology-based cophylogenetic analyses used either a fully resolved parasite ML tree or a parasite ML tree with nodes supported by a bootstrap value under 70 collapsed. Solutions depicted in bold are visualized in Fig. 3.

**Table 4:** Accession numbers of parasite sequences and host vouchers used to reconstruct a combined nuclear-mitochondrial phylogeny of *Cichlidogyrus* infecting Lake Tanganyika tropheine cichlids.

Host species	Country	Locality	RMCA (MRAC) accession numbers (host vouchers)	GenBank accession numbers (parasite sequences)	
				rDNA	COI
‘*Ctenochromis*’ horei*	Tanzania	Mtosi	B2-04-P-117	KT037139-41	KT037337
	Zambia	Kalambo Lodge	B2-04-P-119-131 (1,4,5,6,7,8,9,10,11)	KT037142-65	/
		Mbita Island	B2-04-P-118	KT037166-8	KT037338
‘*Gnathochromis*’ pfefferi*	D.R. Congo	Mtoto	T10-2024	KT037169	KT037339
	Zambia	Kalambo Lodge	B2-04-P-149-165 (1,2,3)	KT037170-3	KT037340-1
Interochromis loocki*	Zambia	Kalambo Lodge	B3-36-P-1	KT037174-5	KT037342-3
		Muzumwa	B1-23-P-339-341 (1,6)	KT037176-81	KT037344-6
Limnotilapia dardennii*	D.R. Congo	Bemba	B0-12-P-1205	KT037182-3	sequence too short for GenBank
	Zambia	Kalambo Lodge	B2-04-P-132-148 (4)	KT037184-5	/
		Muzumwa	T11_Lida5	KT037186	KT037347-8
*Lobochilotes labiatus*	D.R. Congo	Lubumba	B0-12-P-312-315 (2)	KT037187-90	KT037349
	Tanzania	Mtosi	B2-04-P-184-189 (1)	KT037191-2	KT037350
		Musamba	B2-04-P-190-194 (2)	KT037193-4	KT037351
	Zambia	Kalambo Lodge	B2-04-P-166-181 (1,2,3,4,5)	KT037195-230	/
		Mbita Island	B2-04-P-113	KT037231-2	/
*Petrochromis famula*	D.R. Congo	Kikoti	B0-12-P-866	KT037233	KT037352
	Zambia	Mutondwe Island	B2-04-P-195-199 (2,4)	KT037234-8	KT037353-4
*Pe. fasciolatus*	D.R. Congo	Mtoto	B0-12-P-861	KT037239-41	KT037355-7
*Pe. macrognathus*	D.R. Congo	Kapakwe	T10-Pema1	KT037242-4	KT037358-60
		Luhanga	B0-12-P-1206	/	KT037361-3
*Pe. polyodon*	D.R. Congo	Bemba	B0-12-P-1208-1210 (788)	/	KT037364
		Luhanga	B0-12-P-1207	KT037245	KT037365
		Mugayo North	B0-12-P-1211	KT037246	KT037366-7
	Tanzania	Musamba	B2-04-P-200-201 (1,2)	KT037247-51	KT037368
	Zambia	Mbita Island	B2-04-P-211	KT037252	/
*Pe. trewavasae trewavasae*	D.R. Congo	Kikoti	B0-12-P-867, B0-12-P-456-457 (1,2)	/	KT037369-72, one sequence too short for GenBank
*Pe. trewavasae ephippium*	Zambia	Mbita Island	B2-04-P-203-204 (1,2)	KT037253-6	KT037373-4
		Mutondwe Island	B2-04-P-202	KT037257-62	/
northern Pseudosimochromis babaulti*	D.R. Congo	Bemba	B0-12-P-846	KT037263-4	KT037375-7
southern Ps. babaulti*	D.R. Congo	Kikoti	B0-12-P-426	KT037265	KT037378
		Mufazi	B0-12-P-816-829 (3,8)	/	KT037379-80
	Zambia	Kalambo Lodge	B2-04-P-70-91 (1,10)	KT037266-7	KT037381-2
Ps. curvifrons*	D.R. Congo	Bemba	B0-12-P-430	KT037268-70	KT037383-4
		Luhanga	B0-12-P-748	KT037271	/
		Mugayo North	B0-12-P-750	KT037272-3	KT037385-6
	Zambia	Kalambo Lodge	B2-04-P-98-110 (1,4)	KT037274-7	KT037387
		Kasakalawe/Chanzimu	B2-04-P-97	KT037278	KT037388
		Wonzye Point	B2-04-P-95	KT037279	KT037389
Ps. marginatus*	D.R. Congo	Bemba	B0-12-P-429	KT037280-1	KT037390-1
		Lubumba	B0-12-P-379-380	KT037282-92	/
Simochromis diagramma*	Zambia	Kalambo Lodge	B2-04-P-52, B2-04-P-58-64 (1,4,5,6,8)	KT037293-313	KT037392-3
		Mbita Island	B2-04-P-114-115 (2)	KT037314-5	/
*Tropheus annectens*	D.R. Congo	Kapakwe	B0-12-P-1235	KT037316	/
		Mugayo North	B0-12-P-1212-1234 (660,673)	KT037317-20	KT037394-7
*T. brichardi*	Burundi	Nyanza Lac	B0-12-P-1236-1256 (1336,1339,1341,1345,1346)	KT037321-6	KT037398-406
*T. duboisi*	D.R. Congo	Bemba	B0-12-P-7	KT037327-9	KT037407
*T. moorii*	Zambia	Mutondwe Island	B2-04-P-205-210 (1,3)	KT037330-4	KT037408
Astatotilapia burtoni*	Zambia	Mpulungu	T11-CHC2**	/	KT037409-10
Serranochromis robustus jallae*,***	Zambia	Fiwili	B5-15-P-1	KT037335	KT037411-3
*Neolamprologus fasciatus*	Zambia	Kalambo Lodge	B2-04-P-212	KT037336	/

*Parasite species morphologically characterized and type/voucher material available from earlier studies^24^,^25^,^40^,^44^,^86^.

**Also represented in the tissue collection of the Zoological Institute of the University of Basel.

***Parasite voucher in the RMCA invertebrates collection: MRAC 37791.
