# Supplementary material for: Hidden biodiversity in an ancient lake: phylogenetic congruence between Lake Tanganyika tropheine cichlids and their monogenean flatworm parasites
Source: Sci Rep. 2015 Sep 3;5:13669. doi: 10.1038/srep13669 (PMC4558575; doi:10.1038/srep13669)
Supplement: Supplementary methods S1 [file srep13669-s1.pdf]

Supplementary methods S1

Hidden biodiversity in an ancient lake: phylogenetic congruence between Lake Tanganyika tropheine cichlids and their monogenean flatworm parasites

Maarten P.M. Vanhove, Antoine Pariselle, Maarten Van Steenberge, Joost A.M. Raeymaekers, Pascal I. Hablützel, Céline Gillardin, Bart Hellemans, Floris C. Breman, Stephan Koblmüller, Christian Sturmbauer, Jos Snoeks, Filip A.M. Volckaert and Tine Huyse

DNA extraction, amplification and sequencing

DNA extraction was performed following Ziętara et al.[1]. We added 5 µL of a double concentrated lysis solution containing a final concentration of 1x PCR (Eurogentec), 0.45% Tween 20 (Merck), 0.45% NP40 (Calbiochem) and 60 $\mu g.L^{-1}$ proteinase *K* (Sigma). Enzymatic digestion was carried out at 65 °C for 25 min, followed by inactivation of the enzyme at 95 °C for 10 min. As this method did not always prove successful, alternatively the digestion was prolonged to 120 min, intermitted halfway by vortexing the sample, with subsequent inactivation of proteinase for 25 min. Polymerase Chain Reaction (PCR) was performed with a GeneAmp PCR system 2700 thermocycler (Applied Biosystems). The reaction mix contained 2.5 µL of 10x PCR buffer (Eurogentec), 2.5 µL of 2 mM dNTPs (Thermo Scientific), 1 or 0.75 µL of 50 mM $MgCl_2$ (Eurogentec), 0.2 µL of 5 U/µL *Taq* Silverstar Polymerase (Eurogentec), 1 µL of each primer (20 µM) (Eurogentec) and 1 µL of template DNA, topped up with milli-Q H_2O to a total volume of 25 µL. Nuclear primers used were ITS1A (5′-GTAACAAGGTTTCCGTAGGTG-3′) and ITS2 (5′-TCCTCCGCTTAGTGATA-3′)[2]. This combination spans the entire first and second internal transcribed spacers (ITS-1 and ITS-2) and intervening 5.8S rDNA. When unsuccessful, nested PCR was performed using internal primer ITSR3A (5′-GAGCCGAGTGATCCACC-3′)[2]

together with ITS1A, thus amplifying the ITS-1 fragment. Following initial denaturation for 3 min at 96 °C, samples were subjected to 35 or 40 cycles of 50 s at 95 °C, 50 s at 52 °C (50 °C for nested PCR) and 50 s at 72 °C. After a final elongation of 7 min at 72 °C, samples were cooled to 4 °C. To amplify the mitochondrial cytochrome oxidase *c* subunit I gene (COI), the primers ASmit1 (5'–TTTTTTGGGCATCCTGAGGTTTAT–3')[3] and Schisto3 (5'–TAATGCATMGGAAAAAAACA–3')[4] were used, replacing the latter by ASmit2 (5'–TAAAGAAAGAACATAATGAAAATG–3')[3] in a nested PCR when necessary. The composition of the PCR reaction mix was identical to the one used for the nuclear marker, except that the quantity of $MgCl_2$ was kept at 1 µL. The PCR protocol for COI comprised an initial denaturation at 95°C for 5 min, 40 cycles of 1 min at 94°C, 1 min at 50°C and 1 min at 72°C, followed by a final elongation for 7 min at 72°C and cooling to 4°C. PCR products were purified with NucleoFast 96 PCR (Macherey-Nagel) or with a GFX PCR DNA and Gel Band Purification kit (GE Healthcare), according to the manufacturers' guidelines. The Big Dye Terminator v.3.1 sequencing protocol (Applied Biosystems) was applied at 1/8 dilution for sequencing, with the same primers as in the amplification protocol. Products were run on an ABI PRISM 3130 Avant Genetic Analyser automated sequencer (Applied Biosystems).

References

1. Ziętara, M. S., Arndt, A., Geets, A., Hellemans, B. & Volckaert, F. A. M. The nuclear rDNA region of *Gyrodactylus arcuatus* and *G. branchicus* (Monogenea: Gyrodactylidae). *J. Parasitol.* **86,** 1368-1373 (2000).

2. Matějusová, I., Gelnar, M., McBeath, A. J. A., Collins, C. M. & Cunningham, C. O. Molecular markers for gyrodactylids (Gyrodactylidae: Monogenea) from five fish families (Teleostei). *Int. J. Parasitol.* **31,** 738-745 (2001).

3. Littlewood, D. T. J., Rohde, K. & Clough, K. A. Parasite speciation within or between host species? Phylogenetic evidence from site-specific polystome monogeneans. *Int. J. Parasitol.* **27,** 1289-1297 (1997).

4. Lockyer, A. E. et al. The phylogeny of the Schistosomatidae based on three genes with emphasis on the interrelationships of *Schistosoma* Weinland, 1858. *Parasitology* **126,** 203-224 (2003).
